# Comparison of hypoxia transcriptome *in vitro* with *in vivo* gene expression in human bladder cancer

**DOI:** 10.1038/sj.bjc.6602666

**Published:** 2005-07-19

**Authors:** J J Ord, E H Streeter, I S D Roberts, D Cranston, A L Harris

**Affiliations:** 1 Department of Urology, Churchill Hospital, Oxford, UK; 2 Department of Cellular Pathology, John Radcliffe Hospital, Oxford, UK; 3 Institute of Molecular Medicine, Cancer Research UK Laboratory, John Radcliffe Hospital, Oxford, UK

**Keywords:** microarray, hypoxia, carcinoma, bladder, HIF

## Abstract

Hypoxia-inducible genes have been linked to the aggressive phenotype of cancer. However, nearly all work on hypoxia-regulated genes has been conducted *in vitro* on cell lines. We investigated the hypoxia transcriptome in primary human bladder cancer using cDNA microarrays to compare genes induced by hypoxia *in vitro* in bladder cancer cell line EJ28 with genes upregulated in 39 bladder tumour specimens (27 superficial and 12 invasive). We correlated array mRNA fold changes with carbonic anhydrase 9 (CA IX) staining of tumours as a surrogate marker of hypoxia. Of 6000 genes, 32 were hypoxia inducible *in vitro* more than two-fold, five of which were novel, including lactate transporter SLC16A3 and RNAse 4. Eight of 32 hypoxia-inducible genes *in vitro* were also upregulated on the vivo array. Vascular endothelial growth factor mRNA was upregulated two-fold by hypoxia and 2–18-fold in 31 out of 39 tumours. Glucose transporter 1 was also upregulated on both arrays mRNA, and fold changes on the *in vivo* array significantly correlated with CA IX staining of tumours (*P*=0.008). However, insulin-like growth factor binding protein 3 mRNA was the most strongly differentially expressed gene in both arrays and this confirmed its upregulation in urine of bladder cancer patients (*n*=157, *P*<0.01). This study defines genes suitable for an *in vivo* hypoxia ‘profile’, shows the heterogeneity of the hypoxia response and describes new hypoxia-regulated genes.

Invasive bladder cancer has a mortality of approximately 50% and new approaches to treatment include inhibiting angiogenesis or targeting hypoxia cells. We and others have previously shown the importance of angiogenic factors, such as vascular endothelial growth factor (VEGF) measured in the urine or in the tumour, in mediating bladder tumour angiogenesis (Chow *et al*, 1999; Crew *et al*, 1999; Crew *et al*, 2000). We showed striking luminal induction of VEGF in superficial bladder cancer (Turner *et al*, 2002). Vascular endothelial growth factor is a key angiogenic factor and microvessel density as a measure of angiogenesis has been shown to be highly associated with poor outcome in invasive bladder cancer (Bochner *et al*, 1995). Vascular endothelial growth factor is well recognised to be induced by hypoxia via hypoxia inducible factor (HIF) and this suggests that genes induced by hypoxia may be related to the aggressive phenotype. Indeed many proteins upregulated by hypoxia play a role in invasion such as urokinase plasminogen activator receptor, matrix metalloproteinase 2 and cathepsin D. Direct clinical measurements of tumour hypoxia with Eppendorf electrodes have shown it to be independently related to prognosis, being associated with aggressive local growth, metastasis and treatment failure whether from radiotherapy or surgery, although these have not been done in bladder cancer (Brizel *et al*, 1996; Hockel *et al*, 1996; Nordsmark and Overgaard, 2000).

Apart from intratumoural hypoxia, genetic alterations also upregulate HIF; loss of tumour suppressor genes such as PTEN and activation of H-ras induce HIF, and these alterations are common in bladder cancer.

Carbonic anhydrase IX (CA IX) is an extracellular enzyme attached to the cell membrane that may generate the acidic pH of tumours. We previously demonstrated the induction of CA IX by hypoxia, its regulation by HIF and its correlation with tissue hypoxia measured by oxygen electrodes in cervical cancer (Airley *et al*, 2001). We also demonstrated that bladder cancers show widespread staining for CA IX on the luminal surface and perinecrotic areas (Turner *et al*, 2002). Clinical studies also correlated the expression of CA IX with staining of bladder tumours by pimonidazole (Wykoff *et al*, 2000). A recent study also shows that bladder cancers expressing HIF have a poor prognosis independent of stage and grade (Theodoropoulos *et al*, 2004). Thus, hypoxia has a substantial role in bladder cancer biology.

We hypothesised that other hypoxia-regulated genes might be significantly expressed in bladder cancer and relevant to its biology. Since most analysis of genes induced by hypoxia has been on cell lines *in vitro*, we analysed gene expression in primary tumours *vs* comparator cell lines in normoxia. We considered that genes upregulated in our tumour array would include those induced by hypoxia since our comparator cell lines were in normoxia.

We defined a hypoxia-upregulated profile of genes in an invasive bladder cancer cell line (EJ28) and compared the expression of those genes with those upregulated in 39 primary bladder tumours. This contrasts with other expression arrays published for bladder cancer where a panel of four bladder cancer cell lines (Sanchez-Carbayo *et al*, 2003) or four normal bladder tissue samples (Dyrskjot *et al*, 2003) were used as reference. Using the *in vitro* induced genes, we were able to define for each tumour an *in vivo* hypoxia profile. We then stained 33 available tumours from the expression study for CA IX as a surrogate marker of hypoxia and compared the *in vivo* hypoxia gene profile with tumour CA IX staining and necrosis. We also defined a hypoxia-upregulated profile of genes from a single culture of normal urothelial cells cultured from a strip of fresh human ureter. As insulin-like growth factor binding protein 3 (IGFBP-3) was one of the most induced and expressed genes in cancer and normal urothelia *in vitro* and on the *in vivo* array, in a separate set of 157 urine samples from bladder cancer patients and controls we analysed protein levels of IGFBP-3.

## MATERIALS AND METHODS

### Cell culture

Bladder cell lines were cultured in Dulbecco's modified Eagle's medium (DMEM) with HEPES (EJ28, 253J 2T10) or RPMI 1% (RT112) and supplemented with 1 mM glutamine (GibcoBRL, UK), 0.002 mg ml^−1^ puromycin and 10% fetal bovine serum (FBS) (Globepharm, UK), in a humidified atmosphere of 5% CO_2_ at 37^o^C. Human umbilical vein endothelial cells (HUVE cells) were maintained in MCDB 131 (Gibco, Paisley, Scotland, UK). Human umbilical vein endothelial cells were purchased from Clonetics BioWhittaker (Wokingham, Berkshire, UK) and were cultured in MCDB 131 medium (Invitrogen, Paisley, Scotland, UK) containing 20% fetal calf serum (Sigma-Aldrich), 100 U ml^−1^ penicillin, 100 *μ*g ml^−1^ streptomycin, 2 mM glutamine, 5 IU ml^−1^ heparin and 50 *μ*g ml^−1^ endothelial cell growth supplement (Sigma, Dorset, UK). Human umbilical vein endothelial cells were grown on plates coated with 0.2% gelatine, and were used up to the 8th passage. Cells were exposed to hypoxia by incubation in a humidified atmosphere of 0.1% O_2_, 5% CO_2_ and the remainder N_2_ in a NAPCO 7301 incubator (Precision Scientific, USA).

### Normal urothelial culture

Samples of ureter were collected with informed consent and approval from the Local Research Ethics Committee. A strip of ice-cooled fresh normal human ureter from a patient undergoing nephrectomy for renal cell cancer was placed in stripper medium (0.1% EDTA, 10 mM HEPES, Trasylol 500 000 IU in 500 ml Hank's Balanced Salt Solution minus calcium and magnesium) for 6 h at 4°C. The urothelial lining was scraped off, digested with collagenase for 30 min and plated on collagen-coated plates (Becton Dickinson, NJ, USA) and cultured as previously described (Cordon-Cardo *et al*, 1984; Dubeau and Jones, 1987; Schaafsma *et al*, 1989). Cytospins were performed and cells stained for URO5 antibody, Cytokeratin 7 and Cytokeratin 20 confirming urothelium as the cell of origin.

### RNA extraction and storage

RNA was extracted using 7 ml Trireagent (Sigma, UK) per 75 cm^2^ dish. Concentrations of RNA and purity were assessed on a UV spectrometer. The integrity of the mRNA in this total RNA sample was checked by running the RNA on a 1% agarose gel. Tumour samples were collected with informed consent and approval from the Local Research Ethics Committee.

### Sanger microarrays, chips and analysis

Hver 1.2.1 cDNA microarrays were obtained from the Microarray Consortium, Sanger Centre, Hinxton, Cambridge, UK. These arrays contain 9932 spotted samples of cDNA originating from the I.M.A.G.E. collections of the Human Genome mapping Project and Research Genetics, Hinxton, UK, representing approximately 6000 human genes in total. A GSI Lumionics Scan Array 400 array reader with Scanarray and Quantarray® software was used to analyse the slides. The analysed data were imported into GeneSpring® software (Silicon Genetics, USA). Data were normalised internally by region on each chip (regional normalisation) to compensate for systematic variation over the surface of the array. The mathematical Lowess correction was applied to allow for nonlinear fluorescence characteristics of the dyes.

### Generation of cell line reference RNA

To provide a controlled reference for each competitive hybridisation, a panel of 11 cell lines was harvested while in exponential growth in culture (SK23, NCI-H1385, NCI-H69, IM-9, MCF-7, OVCAR-3, MOLT-4, HEPG2, SW620, HT1376, W38). These cell lines were selected according to previously published work at Stanford (Perou *et al*, 2000) in order to reliably provide a constant level of expression over the vast majority of genes on the array.

### Generation of fluorescently labelled single-stranded cDNA and competitive hybridisation of labelled single-stranded cDNA

Between 25 and 50 *μ*g total RNA in 75% ethanol was mixed with 1 *μ*l of bacterial RNA cocktail (as internal control) and applied to the microarray slide. This slide was placed in a hybridisation chamber humidified with hybridisation buffer. Incubation occurred at 47°C for 12–24 h. The slides were then placed in a slide rack and washed in darkness in three different solutions containing SSC and 0.1% SDS on a tilting table for 1 h. Slides were dried by centrifugation.

### Microarray analysis of genes upregulated by hypoxia (0.1%) in cell line EJ28

We analysed gene expression in an aggressive bladder cancer cell line EJ28 under normoxia and 16 and 24 h hypoxia (0.1%). There were four replicates at each time point grown in separate plates compared with normoxic cells from the same passage harvested at the same time.

### Microarray analysis of 39 bladder cancers compared with cancer cell line panel

The labelled cDNA from 39 bladder cancers was hybridised to the chip with labelled cDNA from a panel of 11 cancer cell lines from different types of cancer.

### Real-time reverse transcription–polymerase chain reaction (RT–PCR)

We used a commercial probe and primer sets (Applied Biosystems, USA) for genes of interest. After RT of RNA (Invitrogen), cDNA was diluted down to 0.5 *μ*g *μ*l^−1^. The gene probe had an FAM reporter dye. We chose Beta-2-microglobulin (B2M) as a control gene as this is not known to be upregulated by hypoxia in the literature, and was not upregulated by hypoxia in the cell line on the array (data not shown). The relative Delta Ct technique of real-time analysis was used looking at changes in cycle threshold between three normoxic replicates and three hypoxic replicates. Two-tailed nonparametric *t*-tests (*P*<0.05) were used to analyse the difference between replicates.

### Bladder tumour tissue microarray

A tissue array was made from paraffin blocks of 33 available gene array cases. Whole sections of bladder cancer stained with haematoxylin and eosin were reviewed under light microscopy by a consultant histopathologist (IR) specialising in urological cancer and one researcher (JO). Scoring of whole slides for necrosis (negative=none, positive=either comedo or gross intratumoral necrosis) was done blinded to the array results. Tissue arrays are accepted forms of rapidly assessing antibodies on a panel of tumours. The cores are significantly representative of the whole block so long as at least two cores are used and regional differences in staining are taken into account when selecting cores (Camp *et al*, 2000; Nocito *et al*, 2001). Carbonic anhydrase IX staining is known to be expressed in bladder cancer and to occur more frequently around areas of necrosis (Wykoff *et al*, 2000). We therefore selected two 1 mm cores from representative areas of tumour and if any necrotic areas existed another two perinecrotic cores.

### Carbonic anhydrase IX staining and scoring of tissue array

The tissue array was stained with the CA IX antibody M75 as described (Wykoff *et al*, 2000). Scoring was as described (Turner *et al*, 2002): a score of 0–3 (0=no staining, 3=strong staining in >50% of the core) was given based on intensity and area of membrane staining of tumour cells. Scores from more than one core were summed. Carbonic anhydrase IX positivity was defined as a score >1.5, which was the median score. Correlation of tumour CA IX staining scores with gene fold changes for all eight genes individually and summed was tested using linear regression. Goodness of fit was calculated as *r*^2^ and *P*<0.05 cutoff taken for a significantly nonzero slope.

### Urine collection

Urine was collected with informed consent fresh from patients with histologically confirmed bladder cancer, an average of 1 week before operation as described (Crew *et al*, 1999). There were 69 superficial cancers, 19 invasive, four carcinoma *in situ*, 34 patients who had previously had bladder cancer resected and were now clear, and 29 controls. Of 29 controls, 12 were 43–80 years old who had full investigations for haematuria with no bladder cancer found; the remaining 17 were medical student volunteers aged 18–30 years with no blood in the urine on dipstix. Urine levels of IGFBP3 were compared using a two-tailed *t*-test, nonparametric with significance cutoff *P*<0.05.

## RESULTS

### Array results in bladder cancer cell line

We minimised false positive genes by initially including those with raw absolute values of >1000. Of 6000 genes analysed, 32 (0.53%) were upregulated by hypoxia in the cell line EJ28 more than two-fold in at least six of eight replicates (four at 16 h and four at 24 h) (Table 1). Adrenomedullin (ADM), upregulated 17-fold, and IGFBP-3, upregulated 12-fold, were the most upregulated genes, with VEGF less at 2.5-fold. Adrenomedullin is known to have a range of effects on vasculature including vasodilatation and angiogenesis (Nikitenko *et al*, 2002). Insulin-like growth factor binding protein 3 is the major binding protein for insulin-like growth factors in the blood and also has IGF-independent apoptotic effects. Nearly all genes either increased or retained the upregulation found at 16 h and at 24 h. These genes divided into functional groups typical of hypoxia-induced genes (Harris, 2002) (Table 1). Several genes not previously known to be upregulated by hypoxia included Ribonuclease 4 RNAse4 3.1-fold, monocarboxylate transporter family member 4 (SLC16A3 also called MCT4), Preferentially expressed antigen of melanoma PRAME 4.9-fold and Importin Beta 3 4.1-fold. The protein kinase C substrate MARCKS also appeared upregulated 2.2-fold at 16 h.

### Validation of hypoxia-upregulated genes by RT–PCR in EJ28, bladder cancer cell lines and primary urothelial and endothelial cell culture

Six genes were analysed: IGFBP-3, RNAse4, SLC16A3, MARCKS, using two controls B2M and Peroxiredoxin. Two novel genes not previously known to be upregulated by hypoxia *in vitro* were RNAse 4 and SLC16A3 (a lactate transporter chosen for its potential relevance to hypoxia). Both were confirmed to be upregulated in a panel of cell lines (Table 2). SLC16A3 RNA was also upregulated by hypoxia in HUVE cells but not in cultured normal urothelium. RNAse 4 like IGFBP-3 was upregulated by hypoxia in the cell lines and normal urothelial cells. The control gene B2M showed no significant change by comparison. Nor did a second control gene Peroxiredoxin selected from the tumour array by its lack of fold change (data not shown). MARCKS was upregulated by hypoxia 2.1-fold on the *in vitro* array. However, MARCKS upregulation in the cell line panel was not confirmed.

### Array results in primary bladder tumours

**Figure 1:**
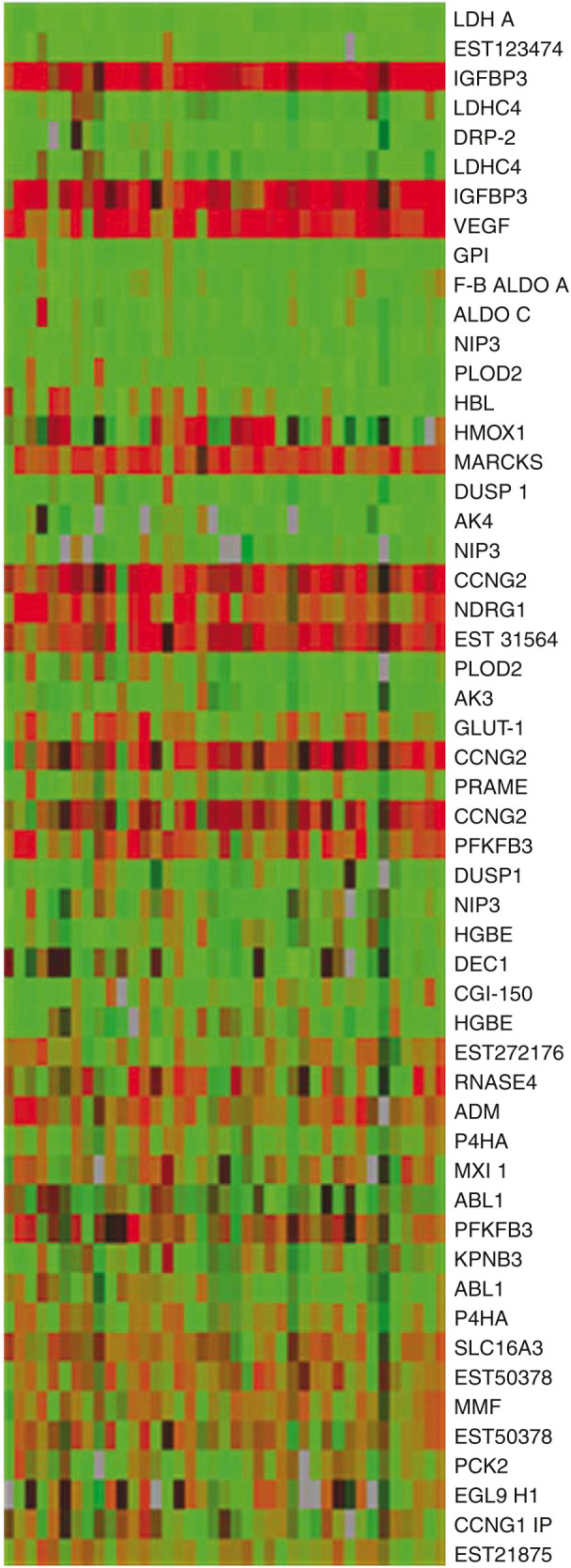
Graphic representation of array results of *in vivo* hypoxia gene expression in bladder cancers, for all genes induced by hypoxia *in vitro* (as listed in Table 1): in order of Genespring ‘Interest’ function that places the most statistically trustworthy and greatest fold changes towards the top of the list. Some genes have more than one result as they were represented by more than one spot on the array. 39 columns=39 tumour samples. Samples 1 (on left) to 26=superficial, samples 27–39=muscle invasive. One row=one gene as labelled. Tumour cDNA was competitively hybridised against a panel of 11 cancer cell lines. Red=two-fold or more upregulation, green=downregulation, brown equals no change. Brightness=statistical trust.

**Figure 2:**
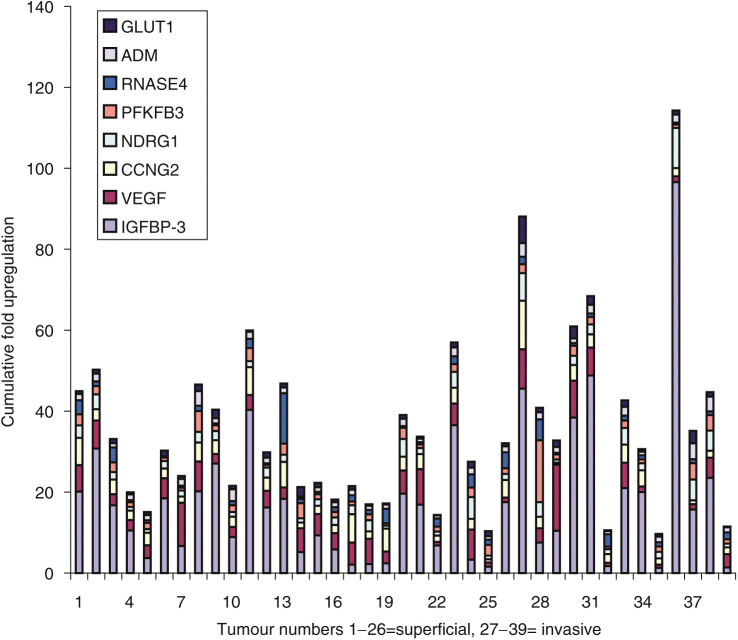
Tumour samples categorised by the fold upregulation of the eight genes upregulated by hypoxia in the cell line and upregulated more than two-fold in more than five tumour samples on the *in vivo* array. Insulin-like growth factor binding protein 3 makes the largest fold contribution and is upregulated more than two-fold in the majority of samples.

Of 6000 genes on the tumour arrays, 70 (1.2%) were upregulated more than two-fold in over 30 tumours. We compared the results for hypoxia-regulated genes from the cell line with the bladder tumour array (Figure 1). Of the 32 genes upregulated by hypoxia *in vitro*, eight genes were upregulated more than two-fold with raw signal intensity >1000 in more than five of the 39 tumours. Vascular endothelial growth factor, which is known to be expressed in bladder cancer, was upregulated 2.5-fold by hypoxia and 2–18-fold in 31 out of 39 tumours making it the second most frequently upregulated hypoxia-inducible gene. The most highly and frequently upregulated gene in both arrays, however, was the mRNA IGFBP-3. This was upregulated up to 12-fold by hypoxia *in vitro* and from 2 to 100-fold in 33/39 tumours compared to the cell line panel. The other six genes with more than two-fold upregulation in more than five tumours arrayed were CCNG2 in 30 tumours, NDRG1 in 18 tumours, PFKFB3 in 17 tumours, RNAse4 in 10 tumours, Adrenomedullin (ADM) in eight tumours and Glucose transporter 1 (GLUT-1) in six tumours. The cumulative fold changes for all eight genes are plotted for each tumour (Figure 2). It can be seen that IGFBP-3 is more upregulated than the others in individual tumours.

Of 32 genes upregulated by hypoxia *in vitro*, 24 were downregulated on the *in vivo* array (definition of downregulated – more than 0.5-fold lower in more than five tumour samples). The most downregulated gene *in vivo* was lactate dehydrogenase. Genes that were most induced *in vitro* were more likely to be upregulated *in vivo*; six of the 10 most hypoxia-induced genes on the *in vitro* array were upregulated in the *in vivo* array *vs* only one of the remaining genes (*P*=0.02 Fisher's exact test six out of 10 *vs* one out of 13).

SLC16A3 was strongly regulated in tumour cell lines and HUVE cells *in vitro* and although signal was low in tumours it was upregulated more than two-fold in seven tumours. A paired *t*-test of the raw data showed significant upregulation (mean control signal 520, mean raw signal 690, ratio 1.3, *P*=0.0003). To see if this occurred in cell lines from other tumour types, two breast cancer cell lines were studied (T47D, MDA 468) and also showed induction (Table 2).

### Scoring of tumours for necrosis and hypoxia

**Figure 3:**
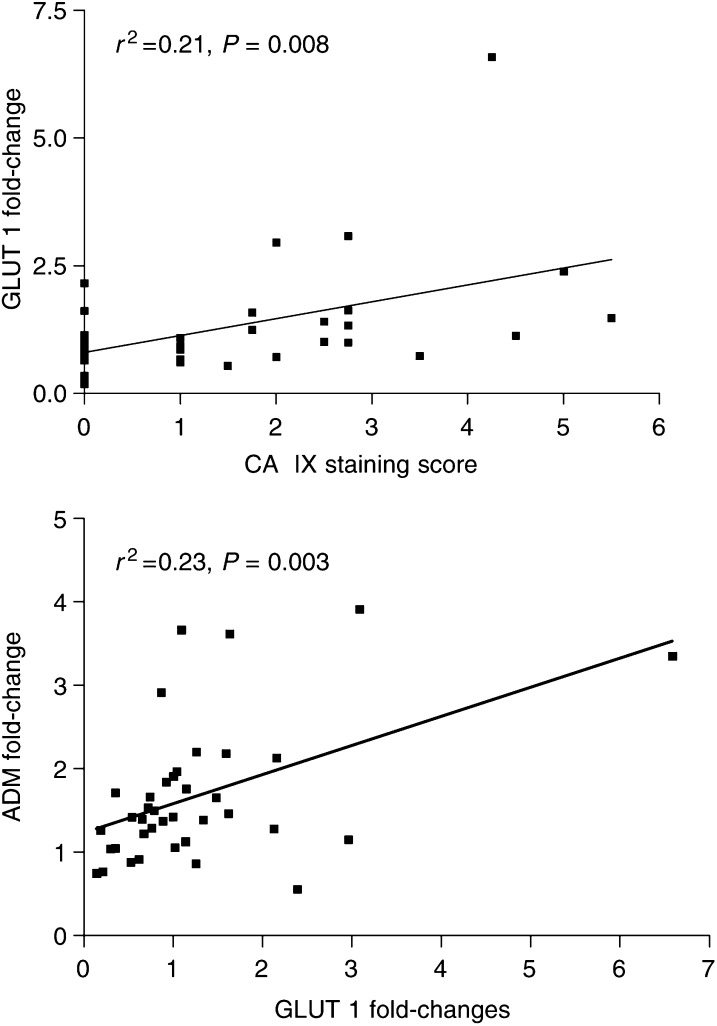
Glucose transporter 1 fold changes *in vivo* correlated with scores for CA IX staining of tumours by immunohistochemistry as a surrogate marker of hypoxia. Adrenomedullin fold changes correlated with GLUT-1 fold changes. Carbonic anhydrase IX itself was not on the array.

To correlate the *in vivo* transcriptome of these tumours with tumour hypoxia, we scored paraffin sections of the tumours using CA IX stain as a marker of hypoxia. We also scored tumours for the presence of intratumoural necrosis seen with haematoxylin and eosin stain. A total of 32 tumours were available for both scores, 11 invasive and 21 superficial. Necrosis was present in five invasives and two superficials. CA IX scoring was positive in three invasive cancers and 13 superficial tumours. A total of three necrotic tumours were also CA IX positive. GLUT-1 fold changes correlated with CA IX scores (*P*=0.008 *r*^2^=0.21, Figure 3), and were significantly higher in CA IX-positive tumours (means 0.9 *vs* 1.8 *P*=0.03 two-tailed *t*-test variances not assumed to be equal).

### Urine IGFBP-3 protein is raised in bladder cancer

**Figure 4:**
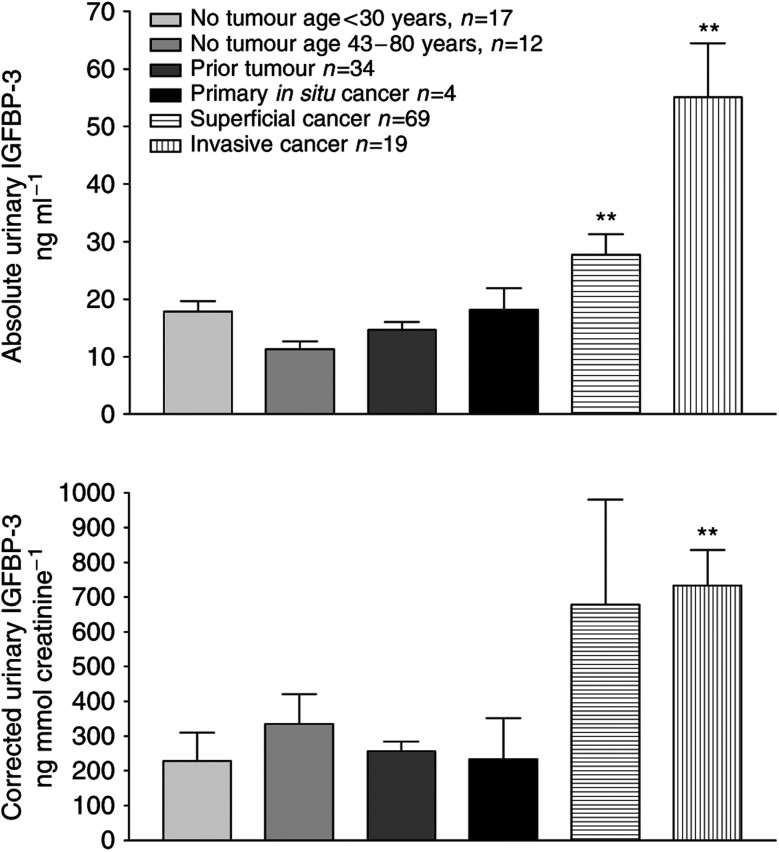
Levels of IGFBP-3 in urine: TOP absolute level, BOTTOM corrected level for urine creatinine. Invasive significantly higher than all control groups in both cases. Superficial significantly higher than control groups for absolute levels only (^**^=*P*<0.01). Error bar=standard error.

Insulin-like growth factor binding protein 3 at the transcript level was upregulated higher and more frequently than VEGF in both *in vitro* and *in vivo* arrays. We therefore measured levels of IGFBP-3 protein in the urine (Figure 4). Urine from 157 bladder cancer patients and controls was collected prospectively and IGFBP-3 levels were measured by ELISA. There was a significant increase in urinary IGFBP-3 concentration from control/clear groups to superficial cancers to invasive cancers, *P*<0.01 for superficial *vs* control/clear, *P*<0.01 for invasive *vs* control/clear (IGFBP-3 ng ml^−1^ mean (standard deviation); controls 15.2 ng ml^−1^ (7.55); clears 14.6 ng ml^−1^ (8.1); superficial 27.7 ng ml^−1^ (29); invasive 55.1 ng ml^−1^ (40)). After correction for urinary creatinine, significance (*P*<0.01) was retained for invasive tumours, but not for the superficial group. There were 15 stage T1G3 tumours in the superficial group. There was no significant difference between levels in the urine of T1G3 tumours and other superficial tumours or with the invasive group. If a single exceptionally high (>4 standard deviations) level in the superficial group is excluded, the mean corrected levels of urine IGFBP-3 increase with stage and grade: superficial (T1G2 or lower) 351 ng mmol^−1^ Cr (creatinine); T1G3 479 ng mmol^−1^ Cr; invasive 733 ng mmol^−1^ Cr.

One of the anonymously taken medical student controls had a level of IGFBP-3 five standard deviations higher than the rest of this group. Despite this, the significant difference between this group and the bladder cancer group held. All medical student control urines dipped negative for blood so an undiagnosed cancer is unlikely in such a young person. The likely explanation is an exercise-induced rise in IGFBP-3 (Manetta *et al*, 2002).

## DISCUSSION

The *in vitro* analysis of hypoxia-induced genes showed several genes already well recognised to be induced by hypoxia, and also other genes likely to be of functional importance in the response to hypoxia.

SLC16A3 is a new hypoxia-regulated gene. This occurred particularly in HUVEC cells, and also in bladder and breast cell lines, but not in cultured normal urothelium. It is a member of the proton-linked transmembrane monocarboxylate transporter (MCT) family with high affinity for lactate (Halestrap and Price, 1999). It is particularly high in the white skeletal muscle, white blood cells and cell lines, suggesting that it may be of particular importance in tissues that rely on high levels of glycolysis for ATP production.

The upregulation of RNAse4 by hypoxia is also novel. The human enzyme is secreted into plasma and the gene (Rosenberg and Dyer, 1995) shares various identical 5′-untranslated regions on its mRNA with the angiogenic factor angiogenin, suggesting that expression of the proteins is under similar control, with obvious implications for their biological activities. Angiogenin is upregulated by hypoxia in cell lines and is raised in the blood and tumours of patients with bladder cancer (Miyake *et al*, 1999).

On the tumour array, 70 of 6000 (1.2%) genes were upregulated more than two-fold in over 30 tumours. Three of these 70 genes (4.3%) were hypoxia inducible on the *in vitro* array (IGFBP-3, VEGF, CCNG2). Since only 32 of 6000 (0.53%) genes on the *in vitro* array were upregulated by hypoxia, hypoxia-induced genes are eight times over-represented in the group of genes most upregulated on the tumour array (*P*< 0.01 *χ*^2^ test). However, of the 32 genes upregulated *in vitro*, only eight were upregulated in the tumour samples.

In contrast, 24 were downregulated more than 0.5-fold in more than five tumour samples (green) *in vivo* (Figure 1). There are several possible reasons for this. The *in vivo* array, comparing as it does tumour samples to a panel of cell lines may be highlighting the more complex influences on gene expression present in the three-dimensional environment. Three-dimensional geometry of *in vitro* cell growth alone has been shown to have dramatic influences on biological function and gene expression profile (Dangles *et al*, 2002).

The apparent downregulation of NIP3, Aldolase C, PLOD2 and other hypoxia-inducible genes on the *in vivo* array in all tumour samples, even in tumours where hypoxia and necrosis were present, may represent a relative basal upregulation of these genes in the monolayer cell culture panel. The subset of known hypoxia-inducible genes that were clearly upregulated *in vitro* but not *in vivo* in general was less inducible. The combination of pure *in vitro* cancer cell populations with basal upregulation of the pathways, possibly via oncogene pathways know to regulate HIF, is probably sufficient to explain this.

Since we used a widely described cell line panel as a control, this has some implications for interpretation of such arrays. In our study we were specifically investigating a defined pathway, but if we used the tumour array results alone without considering genes in the same pathway, this effect may not have been noted.

We might have expected all eight genes significantly upregulated by hypoxia *in vitro* and *in vivo* to correlate with tumour scores of hypoxia and necrosis *in vivo*. Glucose transporter 1 (GLUT1) transcription fold changes on the *in vivo* array showed a significant correlation with the immunohistochemical CA IX score as a surrogate marker of hypoxia. Glucose transporter and ADM also showed significant correlation with each other. Glucose transporter 1 protein immunohistochemistry has previously been shown to correlate with CA IX staining in cervical cancer and, like CA IX, it has been shown to be a marker of tumour hypoxia (Airley *et al*, 2001, 2003). The correlation here shows that the same is also likely to be true for bladder cancer. Adrenomedullin, a secreted protein, may also prove to be useful as a marker of hypoxic tumours in the urine. The other genes did not correlate with tumour hypoxia and necrosis scores and were upregulated in many tumours. The result highlights the complexity and heterogeneity of the hypoxia response, suggesting that the final pattern may be individual.

In addition to the hypoxic regulation of gene transcripts, oncogenic changes specific to bladder cancer, but which are not found in the majority of the cell line panel, may also contribute to their expression. Examples of genes that are mutated in bladder cancer include p53, PTEN and H-ras, which upregulate HIF (Krishnamachary *et al*, 2003). The HER2 oncogene is known to upregulate IGFBP-3, and was upregulated on the *in vivo* array more than two-fold in 22 of 39 tumour samples.

The gene most frequently and proportionally upregulated was IGFBP-3, which is known to be hypoxia inducible in a number of cell lines. We show that this is also true of bladder cell lines and is not specific to cancer, with upregulation in human cultured urothelium (4.6-fold) and HUVE cells (7-fold). Insulin-like growth factor binding protein 3 may act as a direct apoptotic response to hypoxia, and this implies that there are major selective mechanisms *in vivo* to overcome this pathway and suggests that antagonising the IGF1 receptor could have high selectivity in cancer, with endogenous IGFBP-3 synergising with such inhibition.

In contrast to its extracellular IGF-binding role, an intracellular role of IGFBP-3 has been described (Schedlich *et al*, 2003), with phosphorylation increasing nuclear import of IGFBP-3, with release of IGF. The mechanism involves interaction with Importin Beta (Schedlich *et al*, 2003), a gene not previously recognised to be regulated by hypoxia, which was induced by hypoxia in our study (4.1-fold) *in vitro*. Thus, induction of IGFBP3 may have a more complex role then previously reported.

The finding of raised IGFBP-3 in the urine of bladder cancer patients has not been previously noted. *In vitro* experiments on cell growth and IGFBP-3 make it likely that the levels of IGFBP-3 seen here in urine are of clinical significance. Levels as low as 10 ng ml^−1^ and more commonly 30–50 ng ml^−1^ have been shown to have inhibitory effects on growth of neoplastic cells *in vitro* (Martin and Baxter, 1999; Ewton *et al*, 2002). It is often assumed to be growth inhibitory, but IGFBP-3 has a stimulatory effect on the growth of smooth muscle bladder cells *in vitro* (Weinzimer *et al*, 2001). It must also be noted that the ELISA measures both proteolysed fragments and intact IGFBP-3 (Diamandi *et al*, 2000). The rise we measured may reflect increased proteolysis of IGFBP-3 that would make more IGF available to the tumour. However, the array data suggest at least that direct upregulation of mRNA levels of IGFBP-3 occur, with or without increased proteolysis.

Cyclin G2 is known to be regulated by the VHL/hypoxia pathway (Wykoff *et al*, 2000). It is a negative regulator of the cell cycle with peak expression in S phase and it stimulates apoptosis (Bennin *et al*, 2002). Cyclin G2 RNA levels were shown in another array to be expressed at a lower level in stage T2 bladder tumours than in Ta (Dyrskjot *et al*, 2003). Cyclin G2 was also upregulated in Ta tumours compared to normal tissue. This contrasts with the result on the tumour array here, where there was no significant difference in Cyclin G2 fold change from Ta to T2 tumours. This difference may be because our array used a panel of cell lines as reference rather than normal bladder tissue.

Overall, these results show that there is concordance *in vivo* for a subset of hypoxia-induced genes (ADM, GLUT1 and CA IX protein), which may provide a hypoxia signature for analysis of gene array data. Hoskin *et al* (2003) showed that GLUT1 and CAIX expressions in invasive bladder cancer were correlated with hypoxia and outcome of radiation therapy. However, there is also an individual pattern of expression for each patient. Hypoxia-regulated genes are disproportionately upregulated in primary bladder cancer. The induction of putative proapoptotic, cell cycle arrest pathways commonly occurs (IGFBP-3 and CCNG2) and may act to select apoptosis resistance pathways *in vivo*, and therefore a more comprehensive analysis in clinical trials may help refine predictions of those who would benefit from modulation of hypoxia and the variability in outcome even when using GLUT1.

## Figures and Tables

**Table 1:** Mean fold changes of all 32 genes upregulated more than two-fold by hypoxia (0.1%) at 16 and 24 h in cell line EJ28

	**Fold change at hypoxia**			
**No.**	**16 h**	**24 h**	**Gene**	**Functional group**
*Known to be hypoxia upregulated in other cell lines*				
1	3.4	17	Adrenomedullin (ADM)^*^	Angiogenesis
2	2.6	12.1	Insulin-like growth factor binding protein 3 (IGFBP-3)^*^	Apoptosis
3	2.9	8.2	n-myc downstream regulated protein 1 (NDRG1)^*^	Cell cycle
4	5.3	6.6	NIP3	Proapoptotic
5	2.7	4.7	Induclble 6-phosphofructo-2-klnase (PFKFB3)^*^	Glucose metabolism
6	2.4	4.7	Lysyl hydroxylase (PLOD2)	Tissue remodelling
7	2.2	4.4	MAX interacting protein 1	Transcription factor
8	3.1	4.2	Glucose transporter 1 (GLUT 1)^*^	Glucose metabolism
9	2.3	3.9	1,4-Alpha glucan branching enzyme	Glucose metabolism
10	2.2	3.8	Cyclin G2^*^	Cell cycle
11	2.5	3.7	Proline-4-hydroxylase (P4HA1)	Oxygen sensing
12	3.2	3.2	Adenylate kinase 4	Energy metabolism
13	2.9	3.1	Aldolase C	Glucose metabolism
14	1.9	3	Prolyl-4-hydroxylase alpha subunlt	Oxygen sensing
15	1.8	2.6	Haem oxygenase 1	Haem degradation
16	2.5	2.5	EGL9 homologue 1	Oxygen sensing
17	2.1	2.5	Testis-specific lactate dehydrogenase	Acid base
18	1.9	2.5	VEGF^*^	Angiogenesis
19	2.4	2.5	Adenylate kinase 3	Energy metabolism
20	1.7	2.4	Dual specificity phosphatase 1 (DUSP1/CL100/MKP1)	Cell signalling
21	1.7	2.1	DEC 1	Differentiation
22	2.6	2.1	Glucose-6-phosphate isomerase	Glucose metabolism
23	2.1	2.1	Fructose-bisphosphate aldolase A	Glucose metabolism
				
*Not previously known to be hypoxia regulable*				
24	2.8	8.2	Solute carrier family 16 member 3 (SLC16A3)	Acid base
25	4.5	4.9	Preferentially expressed antigen of melanoma (PRAME)	Antigen
26	2.8	4.1	Importin Beta 3	Nuclear pore protein
27	1.9	3.1	RNAse4^*^	Antimicrobial?
28	2.2	2.9	Protooncogene ABL1	Cell cycle
				
*ESTs*				
29	3	3.9	50378_A	EST
30	3.2	3.3	50022_A	EST
31	3.5	3.3	31564_B	EST
32	2.1	2.4	21875 A	EST

Microarray result of eight biological replicates; genes had to be upregulated more than two-fold with high raw signal intensity (>1000 units) in more than six of eight replicates to avoid false positives and be included in this list. Eight asterisked genes (^*^) were also significantly upregulated on the *in vivo* array. EST=expressed sequence tag.

**Table 2:** Fold changes of IGFBP-3, RNAse4 and SLC16A3 by hypoxia (0.1%) in bladder cancer cell lines, normal cultured human urothelia and HUVE cells analysed by real-time RT–PCR

**Cell type**		**Hours of hypoxia**	**IGFBP-3**	**Control**	**RNAse4**	**Control**	**SLC16A3**	**Control**
Bladder cell line	EJ28	16			3.6	1	5.35	1
								
	EJ28	24	5.5	1.2	6.5	1	6.4	1
								
	2T10	16	14.5	1.3	3.4	1	5.5	1
								
	253J	16	3.4	1.1	3.9	1.1	2.1	1.1
								
	RT112	16	38	1.4	3.8	1.3	3.8	1.4
								
								
Normal urothelium		16	4.6	1.1	3.7	1	−1.3	1
								
Endothelial cells	HUVEC	16	7	−1.4			64	1.6

Three replicates of each experiment were performed and if replicates were more than 0.5 cycles different, the result was not accepted. The control gene Beta-2-microglobulin in each experiment did not differ significantly from normoxia to hypoxia but any trend to change of the control gene is demonstrated by minimal fold changes (range −1.4 to 1.6). Fold change calculated on the basis of 2 to the power of the change in cycle number at a set threshold. Differences in gene of interest in normoxia and hypoxia all showed a significant difference in average cycle threshold (*P*<0.01) unless indicated. Negative values indicate downregulation.
